# Mechanical Properties and Failure Behavior of 3D-SiC_f_/SiC Composites with Different Interphases

**DOI:** 10.1155/2020/6678223

**Published:** 2020-12-09

**Authors:** Deng-hao Ma, En-ze Jin, Jun-ping Li, Zhen-hua Hou, Jian Yin, Xin Sun, Jin-ming Fang, Xiao-dong Gong, Li-na Huang

**Affiliations:** ^1^Science and Technology of Advanced Functional Composite Materials Laboratory, Aerospace Research Institute of Materials & Processing Technology, Beijing 100076, China; ^2^Jiangxi Jiajie Xinda New Materials Technology Co., Ltd, Nanchang 330096, China; ^3^State Key Lab of Powder Metallurgy, Central South University, Changsha 410083, China

## Abstract

Continuous silicon carbide fiber-reinforced silicon carbide ceramic matrix composites (SiC_f_/SiC) are promising as thermal structural materials. In this work, the microstructure and static mechanical properties of 3D-SiC_f_/SiC with PyC, SiC, and PyC/SiC and without an interface prepared via polymer infiltration and pyrolysis (PIP) were investigated systematically in this paper. The results show that the microstructure and static mechanical properties of SiC_f_/SiC with an interphase layer were superior to the composites without an interlayer, and the interface debondings are existing in the composite without an interphase, resulting in a weak interface bonding. When the interphase is introduced, the interfacial shear strength is improved, the crack can be deflected, and the fracture energy can be absorbed. Meanwhile, the shear strength of the composites with PyC and PyC/SiC interfaces was 118 MPa and 124 MPa, respectively, and showing little difference in bending properties. This indicates that the sublayer SiC of the PyC/SiC multilayer interface limits the binding state and the plastic deformation of PyC interphase, and it is helpful to improve the mechanical properties of SiC_f_/SiC.

## 1. Introduction

SiC_f_/SiC have become the most promising structural materials for long-time high-temperature components in aerospace, owing to their excellent physical and mechanical characteristics including lower density, weaker activation, more sufficient fracture toughness, more thermostable performances, better creep resistance, and higher corrosion resistance than that of metal materials [1–8]. It has been recognized that the fiber/matrix interphase has significant effort to the mechanical properties of continuous fiber-reinforced ceramic matrix composites (CFCMCs), which can deflect the matrix microcracks propagation, enable efficient load transfer between fiber and matrix, and protect the fibers [9–14].

At present, the interfacial layers have been developed with a variety of interfacial structures, different preparation techniques, and multiple components, specifically including weak bounding interphase, a strong bounding and layered structure interphase, a strong bounding and (X/Y) N alternating multilayer interphase, and porous structure interphase [15–17]. Studies have shown that pyrolytic carbon (PyC) interface is the most commonly used interface to improve the mechanical properties of SiC_f_/SiC composites due to its typical layered structure and good chemical compatibility with SiC, but the oxidation resistance of PyC interface was poor, which restricted its use in high-temperature environment. Thus, in order to improve the comprehensive mechanical properties of SiC_f_/SiC, the SiC or BN interface layer is usually deposited on the surface of the PyC interface layer [18–20]. Zhou et al. [21] prepared the alternating (PyC/SiC)n multilayer interface layers on the surface of SiC fibers via chemical vapor deposition. It is found that the fracture toughness of the composites with multilayer interface layers is 101% higher than that without interface layer, and the SiC sublayer can effectively prevent PyC interphase from being oxidized. Yang et al. [22] studied the effect of (PyC/BN)n on the mechanical and oxidation properties of the 3D SiC_f_/SiC composites and found that BN can be oxidized into B_2_O_3_ at high temperature, which plays a self-healing role on the crack and protects PyC interface layer from oxidation. It can be seen that a reasonable fiber surface design and introduction of an appropriate interface layer can give full play to the effect of fiber reinforcement and further improve the performance of composite materials. However, due to the complex mechanism of the influence of interface state on the mechanical properties of composite materials, precise quantitative analysis is still lacking. In particular, the influence of interface phase structure on the failure behavior of 3D-SiC_f_/SiC composite materials needs to be further studied.

In this work, three interfacial phases of PyC, SiC, and PyC/SiC were prepared by chemical vapor deposition on the surface of SiC fiber, respectively, and then 3D SiC_f_/SiC with different interfacial phases were fabricated by polymer infiltration and pyrolysis (PIP). The crack propagation and deflection were systematically analyzed in combination with the mechanical properties and the fracture morphology of the composites, and the differences of composite properties and failure behaviors were determined. Meanwhile, the relationship among interfacial shear strength, fracture toughness, and flexure strength of composites was studied systematically.

## 2. Experimental Procedures

### 2.1. Sample Preparation

In this work, the KD-IISiC fibers which were used as the reinforcement and polycarbosilane (PCS) were provided by the National University of Defense Technology (China). Typical parameters of the KD-II SiC fiber are shown in Table 1. The fiber volume fraction of the 3D SiC fiber preforms which were braided by Yixing Tianniao High Technology Co. Ltd., China, is about 40%.

**Figure 1:**
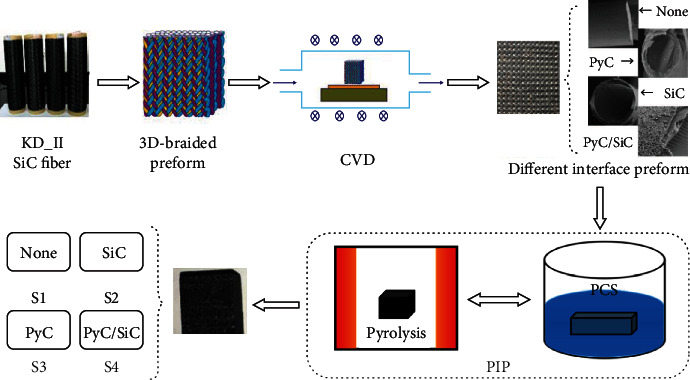
Schematic diagram of the 3D SiC_f_/SiC with various interphases.

Firstly, the SiC fiber preforms were desized at 800°C in vacuum for 30 min, and then, the different interphases (PyC, SiC, and PyC/SiC) were deposited on the surface of the SiC fiber via chemical vapor deposition (CVD). The specific thickness is 0.17~0.35 *μ*m, 0.20~0.45 *μ*m, and 0.53~0.75 *μ*m, respectively. At last, the SiC fiber-reinforced SiC matrix, which was prepared using the precursor infiltration and pyrolysis (PIP) method, was fabricated with different interphases, and marked as follows: S1 (no interface), S2 (SiC), S3 (PyC), and S4 (PyC/SiC), respectively. The specific preparation process is shown in Figure 1.

### 2.2. Sample Characterization

The fracture surfaces after the three-point bending test and polished cross-sections of the samples were observed by field emission scanning electron microscopy (FE-SEM, Hitachi SU-8000). The Instron 5565-5kN multifunctional fatigue tester was used to test the flexural strength and fracture toughness of the samples. The equipment range is 0~50 kN, load error ≤ 1%, and displacement error ≤ 1%s.

## 3. Results and Discussion

### 3.1. Microstructure of the SiC/SiC with Different Interface Layers

**Figure 2:**
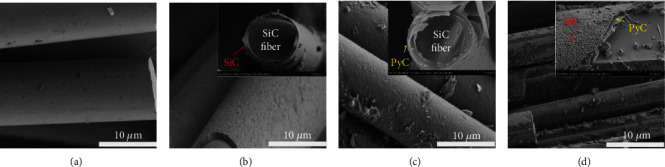
The SEM of the SiC fiber with different interface layers: (a) S1, none; (b) S2, SiC; (c) S3, PyC; (d) S4, PyC/SiC.

The typical microstructure of the SiC fiber with various interface layers, such as PyC, SiC, and PyC/SiC, is shown in Figure 2. Figures 2(b) and 2(c) display the SiC fiber surfaces which are covered by the dense SiC and PyC interfacial layers, respectively, which are deposited via CVD.  Figure 2(d) shows the SiC fiber with the PyC/SiC interface layer. It can be seen clearly that the SiC sublayer, which is deposited on the surface of PyC interface layer, is a thin layer with a SiC nodular interfacial phase. The main reason is that SiC grows mainly in an island pattern within a certain thickness range. In addition, it is found that the morphology of the SiC interface layer on the surface of SiC fiber and pyrolytic carbon substrate is significantly different, which may be due to the different lattice matching degree, surface energy, deposition time, and so on.

**Figure 3:**
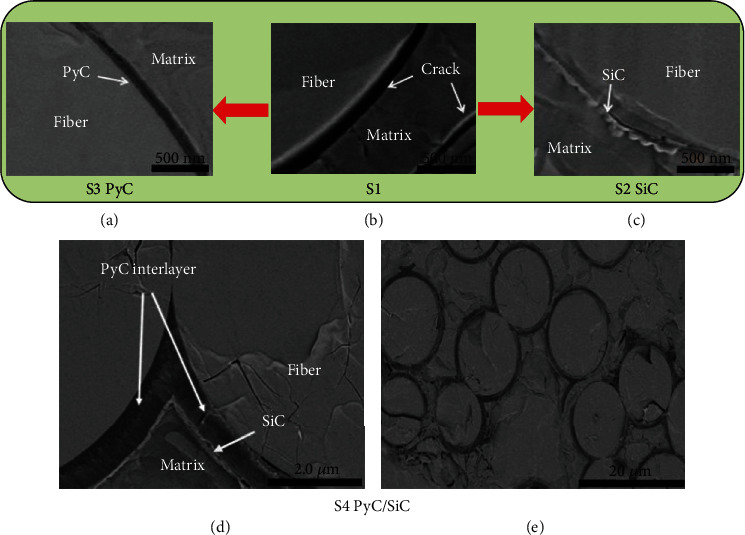
The cross-section morphology of SiC_f_/SiC with different interface layers.

The cross-section morphologies of SiC_f_/SiC composites with different interface layers were characterized by a scanning electron microscope, as shown in Figure 3. Figure 3(b) shows the microstructure of the S1 composite without interface, and it can be found that there is obvious interface debonding in the matrix and fiber. This is mainly due to the mismatch of thermal expansion coefficient between matrix and fiber and the volume effect of matrix cracking [23]. The S3 sample has a dense and uniform PyC interface, as shown in Figure 3(a), and the overall microstructure of the material is relatively complete. For S2 sample, the SiC interface layer is tightly surrounded on the surface of SiC fiber, as shown in Figure 3(c), and it can be found that the boundary has slightly nodular morphology by careful observation. For S4 composite, as shown in Figures 3(d) and 3(e), it can be clearly seen that the fiber surface is tightly wrapped by the PyC first layer and the SiC sublayer. By comparing the morphologies of SiC_f_/SiC composites with different interfaces, it can be found that the introduction of the interface layer can effectively alleviate the stress concentration between fiber and matrix and improve the microstructure of composites.

### 3.2. Mechanical Properties and Failure Behavior of SiC_f_/SiC with Different Interfacial Phases

**Figure 4:**
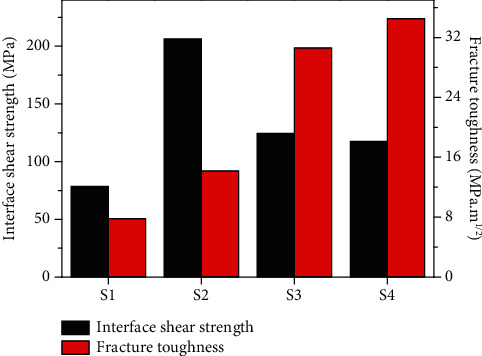
The interface shear strength and fracture toughness of the composites with different interface layers.

**Figure 5:**
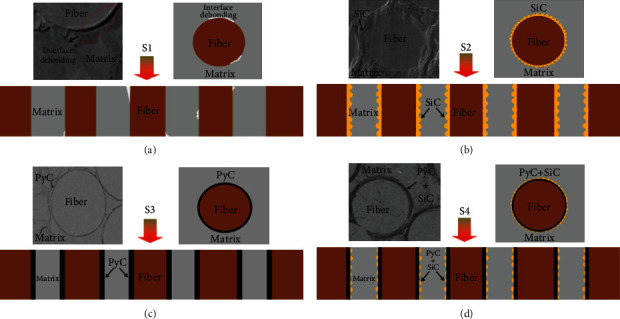
The schematic diagram of single fiber push-out test of the composites with different interface layers.

Figure 4 shows the interfacial shear strength and fracture toughness of the composites with different interfacial layers, in which the interface shear strength is S2 (SiC) > S3 (PyC) > S4 (PyC/SiC) > S1 (no interface). The results indicate that the interfacial layer is beneficial to enhance the interfacial bonding strength of the composites.  Figure 5 shows the schematic diagram of the single fiber push-out test. For the S1 sample, the fiber and matrix appear debonding, as shown in Figure 5(a), and the interface shear strength is the weakest. Due to the high modulus characteristics of CVD SiC and its high chemical compatibility with SiC fiber surface, the S2 sample has the highest interfacial shear strength. Both S3 and S4 samples have PyC interfacial layer, which effectively alleviates the thermal stress caused by the shrinkage of fiber and matrix due to cooling. At the same time, the PyC has a layered crystal structure, and the shear strength is maximum only when the interface thickness is a critical value (about 220 nm). When the PyC interface exceeds the critical thickness, the internal slip probability increases, which will weaken the interfacial shear strength of the composite. The interface thickness of PyC in S4 sample is 2~3 times of that in the S3 sample, but there is not much difference in the interface shear strength between the S3 and S4 samples, indicating that the sublayer SiC interphase introduced in S4 sample is conducive to improving the overall interface bonding strength of the composite.

**Figure 6:**
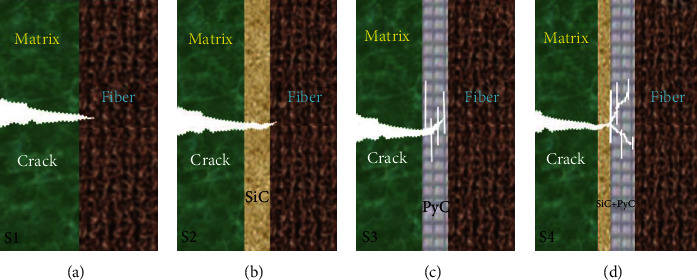
The simulation diagram of crack propagation of the composites with different interface layers.

Meanwhile, the fracture toughness of the composite is S4 (PyC/SiC) > S3 (PyC) > S2 (SiC) > S1 (no interface), in which S1 shows the lowest fracture toughness of only 7.8 MPa m^1/2^. This indicates that the relative crack propagation process is different in different interfaces. For the S1 and S2 samples, when the main crack in the matrix extends to the fiber, the crack tip stress directly acts on the surface of the fiber, leading to the destruction of the fiber, as shown in Figures 6(a) and 6(b). For the S3 sample, due to the introduction of PyC interface, its unique layered structure can help alleviate crack tip stress and effectively induce crack deflection to generate multiple microcracks and absorb more fracture energy, as shown in Figure 6(c). For the S4 sample, the interphase includes the two-phase interface layer of PyC and SiC, in which the crack is more prone to deflection, resulting in a longer propagation path and higher fracture energy absorption, as shown in Figure 6(d). Therefore, M3 has the highest fracture toughness.

**Figure 7:**
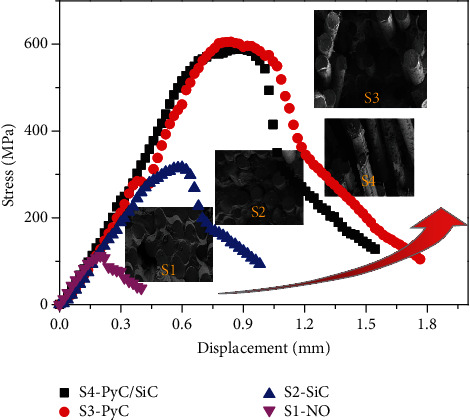
The flexural stress-displacement curves of the composites with different interface layers.

Figure 7 shows the flexural stress-displacement curves of the composites with different interface layers. It can be seen that S1 shows very low bending load and fracture displacement, and the fracture surface is relatively flat. This indicates that S1 composite absorbs little fracture energy during the fracture process, and the reinforcing and toughening effect of the fiber is not effectively exerted. S3 has the highest bending strength and has a significant fiber pull-out. At the same time, a small amount of debris remained on the fiber surface, indicating that the effective bifurcation and deflection occurred when the crack extended to the interfacial layer and absorbed a large amount of fracture energy. The failure behavior of S2 shows small deformation and obvious reduction of bending strength, but it is higher than that of S1, and a small amount of shorter fibers pulls out. This is mainly related to the excessive bonding between the fiber and matrix caused by the SiC interface, and the stress field at the crack tip cannot be effectively released at the interface, resulting in brittle fracture. For S4, its bending behavior is almost the same as that of S3, and it shows a high bending strength and large fracture displacement. Miller et al. found that the thickness of PyC has a significant influence on the flexure strength of SiC/SiC [24]. When the thickness of PyC is 0.13 *μ*m, the bending strength reaches a maximum of 420 MPa, and when the thickness of PyC is 0.58 *μ*m, the bending strength decreases sharply to 275 MPa. This is mainly due to the increase of defects in the interface layer with the increase of PyC thickness. However, the flexure strength and fracture toughness of S3 and S4 samples were similar. The results show that, compared with the single PyC interface, the sublayer SiC interface plays a certain constraint role on the PyC interface layer, which effectively reduces the overall internal slip and defects of the PyC/SiC multilayer interface and thus improves the performance of the composite.

**Figure 8:**
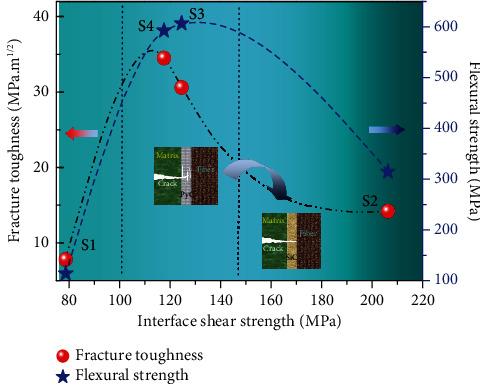
The relationships of the interface shear strength and fracture toughness, flexural strength of the composites with different interface layers.

Figure 8 shows the relationships of the interface shear strength and fracture toughness, flexural strength of the composites with different interface layers. The results show that the fracture toughness and bending strength of the composite first increase and then decrease with the increase of interfacial shear strength, indicating that the moderate interfacial bond strength could effectively improve the fracture toughness and mechanical properties of the composite, which is consistent with the literature reports. At the same time, it is found that the fracture toughness of the composite decreases and the bending strength increases with the increase of the interfacial bonding strength in the area with moderate interfacial bonding strength. In conclusion, the bending properties of composite materials can be further improved through optimizing the interface phase and adjusting the interface bonding strength and fracture toughness. Meanwhile, combined with the microstructure of the interfacial phase, it can be inferred that the mechanical properties of the composites are not only affected by the interfacial bond strength and fracture toughness but also closely related to the surface roughness and shear strength of the interfacial phase itself. Therefore, in order to obtain composites with excellent mechanical properties, in addition to improving the interfacial bonding strength between fiber-interfacial phase and interfacial phase matrix, an appropriate interfacial phases should be designed to enhance their shear strength, absorb higher fracture energy, and so as to improve the fracture toughness.

## 4. Conclusions

1. The order of the fracture toughness of different 3D-braided SiC_f_/SiC is S4 (PyC/SiC) > S3 (PyC) > S2 (SiC) > S1 (no interphase), and the order of the interface shear strength is S2 (SiC) > S3 (PyC) > S4 (PyC/SiC) > S1 (no interphase). The interfacial shear strength not only depends on the state and interaction of fiber-interfacial phase and interfacial phase matrix but also is closely related to the properties of interfacial phase materials, including surface roughness and shear strength
2. The introduction of PyC or PyC/SiC interphase layer is helpful to enhance the interfacial bonding state of the composites and the transition from brittle fracture mode to ductile fracture mode
3. Combined with the microstructure and mechanical properties, it was found that the thickness of the PyC of the PyC/SiC multilayer interface was about 2-3 times that of the single PyC interphase layer. However, the shear strength of the two interfaces was 118 MPa and 124 MPa, respectively, showing little difference in bending properties. This indicates that sublayer SiC is beneficial to enhance the interface bonding strength of PyC/SiC multilayer interface and improve the mechanical properties of materials.

## Figures and Tables

**Table 1:** Parameters of SiC fiber.

SiC fiber type	Diameter (*μ*m)	Density (g/cm^3^)	Tensile strength (GPa)	Tensile modulus (GPa)
KD_II	11-13	2.66	1.8-2.5	270

## Data Availability

The data can be obtained from the article diagram.
